# Efficient engineering of chromosomal ribosome binding site libraries in mismatch repair proficient *Escherichia coli*

**DOI:** 10.1038/s41598-017-12395-3

**Published:** 2017-09-26

**Authors:** Sabine Oesterle, Daniel Gerngross, Steven Schmitt, Tania Michelle Roberts, Sven Panke

**Affiliations:** Department for Biosystems Science and Engineering, ETH Zürich, Mattenstrasse 26, 4058 Basel, Switzerland

## Abstract

Multiplexed gene expression optimization via modulation of gene translation efficiency through ribosome binding site (RBS) engineering is a valuable approach for optimizing artificial properties in bacteria, ranging from genetic circuits to production pathways. Established algorithms design smart RBS-libraries based on a single partially-degenerate sequence that efficiently samples the entire space of translation initiation rates. However, the sequence space that is accessible when integrating the library by CRISPR/Cas9-based genome editing is severely restricted by DNA mismatch repair (MMR) systems. MMR efficiency depends on the type and length of the mismatch and thus effectively removes potential library members from the pool. Rather than working in MMR-deficient strains, which accumulate off-target mutations, or depending on temporary MMR inactivation, which requires additional steps, we eliminate this limitation by developing a pre-selection rule of genome-library-optimized-sequences (GLOS) that enables introducing large functional diversity into MMR-proficient strains with sequences that are no longer subject to MMR-processing. We implement several GLOS-libraries in *Escherichia coli* and show that GLOS-libraries indeed retain diversity during genome editing and that such libraries can be used in complex genome editing operations such as concomitant deletions. We argue that this approach allows for stable and efficient fine tuning of chromosomal functions with minimal effort.

## Introduction

Modifying ribosome binding site (RBS) strength is a popular and efficient approach to tune gene expression levels in prokaryotic systems^1,2^. Small changes in as few as 6 to 8 bp in a spatially well-defined region result in the up- or down-regulation of translation^3^. Since the translation initiation rate (TIR) of any RBS can be approximately predicted solely from its nucleotide sequence^3^, RBS engineering is a useful tool for rationally optimizing a broad range of artificial functions, such as the signalling characteristics of genetic circuits^4^ or pathway fluxes for production of valuable products^5^. Still, in many cases the required level of translation is not known nor is the prediction of the RBS strength exact. In addition, confounding factors such as metabolic burden, essentiality of involved genes, side product formation and toxicity may apply^6^, and predicted expression levels may not be met due to a lack of availability of free ribosomes^7^. Therefore, a library approach is often needed to optimize the TIR for each of the involved genes. As full randomization of even a short part of the RBS produces a set of sequences that is too large to evaluate for a single gene, let alone for gene combinations, and in addition is heavily biased towards non-functional or weak RBSs^8^, a number of tools exist that use the mentioned approximate prediction of RBS strengths to design smaller libraries with a high level of functional sequences whose predicted TIRs still span the entire accessible range. They include the “RBS library calculator”^5,9^, the “MAGE Oligo Design Tool” (MODEST)^10^, the “Empiric Model and Oligos for Protein Expression Changes” (EMOPEC)^11^, and “Reduced Libraries” (RedLibs)^8^.

In many cases, in particular those in which genetic stability of engineered strains is of importance, such as for industrial biotechnology^12,13^, the relevant functions that need tuning are located on the chromosome, presenting a considerable practical obstacle to efficient engineering^14^. However, the development of multiplex automated genome engineering (MAGE), by which small changes in the prokaryotic genome can be efficiently introduced using the lambda red-supported exploitation of single stranded DNA oligonucleotides as fake Okazaki fragments^2,15^, has allowed extending RBS engineering to chromosomal genes, including large scale multiplexing^2,5,16,17^. However, the mechanism of action of this targeted mutagenesis method inherently leads to effects that depend on the sequence of the mutagenic oligonucleotide: the target cell counters mutagenesis by removing mismatches during replication using its MMR enzyme MutS, and the efficiency of this process depends on the length and nature of the mismatch^2,15,18–20^. Consequently, the members of libraries of mutagenic oligonucleotides will meet with quite different fates upon transformation of the target cell. The effect can be avoided in MMR-deficient (MMR^−^) strains, but this results in an increased overall mutation rate (approximately four unwanted point mutations in the genome per MAGE cycle^21–24^ (see also Supplementary Fig. S1)) and can undermine selection (see below). The MMR system can be temporarily inactivated^22–24^, thus limiting the potential for undesired mutations, but this adds additional complexity to the process.

**Figure 1:**
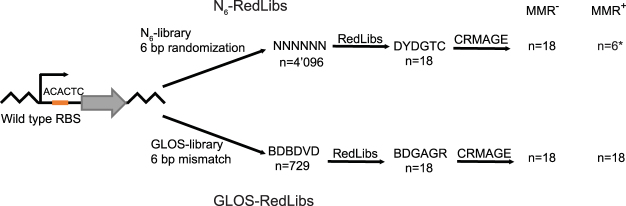
GLOS-RedLibs-supported library design. Schematic comparison of RBS-library design without (upper path) and with (lower path) GLOS-pretreatment. Upper path: A fully randomized RBS sequence (N_6_-library) is reduced by the RedLibs algorithm in order to identify the partially degenerate oligonucleotide sequence (N_6_-RedLibs) that encodes a given number, here n = 18, of RBS sequence variants whose predicted TIRs are as uniformly as possible distributed through TIR space. If this library is used to change the RBS of a target gene in an MMR^−^ strain, all 18 sequences can be expected to be integrated. In an MMR^+^ strain, only 6 (*) sequences would be incorporated assuming that C:C mismatches are not recognized by MMR system^15,18^. For simplicity, we assumed here a repair “cutoff” of 3 bp. In reality, there is no “cutoff” and mismatches of increasing length are repaired with decreasing efficiencies. Lower path: According to the GLOS-rule, only sequence variations that ensure a mismatch of 6 contiguous nucleotides are allowed (a 729-members subset of the 4’096-members fully randomized library of the upper path, GLOS-library). This subset is then reduced via RedLibs to yield the oligonucleotide that encodes n = 18 RBS sequence variants (GLOS-Red-Libs) whose predicted TIRs are most uniformly distributed through TIR space. As the 6 bp mismatches are not recognized by the MMR system, the diversity of the integrated libraries should be the same in MMR^−^ and MMR^+^ strains. B, D, R, V, Y: placeholders for 2 or 3 specific nucleotides, see IUPAC nucleotide code^41^.

Here, we develop an alternative protocol for genome engineering that allows ignoring the MMR system altogether and thus [i] is optimally suited for manipulations in practically relevant MMR^+^ strains and [ii] eliminates the potential for sequence-based bias from library strategies. Specifically, MutS does not recognize insertions or mismatches that are greater than 5 bp^25^. Therefore, using oligonucleotides that introduce the desired mutation(s) on an (at least) 6 bp-long mismatch should eliminate sequence bias. Practically speaking, this rule, which we term the GLOS rule (for genome library optimized sequences) can be easily implemented as an additional requirement for available oligonucleotide selection algorithms, in our case for RBS engineering using the RedLibs algorithm^8^ (Fig. 1).

For practical implementation, we combine our MAGE-based library designs with CRISPR/Cas9-based counter-selection (CRISPR-optimized MAGE or CRMAGE^26^), which has been shown to deliver excellent allelic replacement (AR) efficiencies (more than 95% and 60% for inserting 1 and 6 bp mismatches, respectively^26^). The GLOS-strategy fits well to this approach, as by design all oligonucleotides will have the same mismatch length and thus AR efficiency and gRNA/target binding should be similar, avoiding additional sources of biases (Supplementary Table S1).

Here, we demonstrate the scope of the GLOS approach with a particular focus on avoiding the introduction of sequence bias into library approaches. We validate the GLOS method by modulating the RBS of *E. coli*’s chromosomal *lacZ* gene, and then illustrate its practical utility by modulating a production pathway, specifically the production pathway for the industrially produced vitamin riboflavin (vitamin B_2_, Supplementary Fig. S2)^27^.

## Results and Discussion

### GLOS allows unbiased and efficient sampling of the functional space by oligonucleotide-directed mutagenesis in an MMR^+^ strain

While it was shown previously that both the length and nature of mismatches affect the frequency of repair in MMR^+^ strains^2,15,18–20^, we wanted to confirm that this has indeed a strong impact on library diversity. We chose translation from the chromosomal *lacZ* gene as a test case, as the effects on translation are easy to analyse and LacZ is not essential. Therefore, the AR frequency would not be strongly influenced by changes in expression level, as it would be the case for essential proteins (e.g. due to possible growth defects due to low expression levels). Moreover, it was previously shown that *lacZ* can be modified with high AR efficiency^28^. This was important to exclude variations in AR efficiency due to the position on the genome^10,29^.

**Figure 2:**
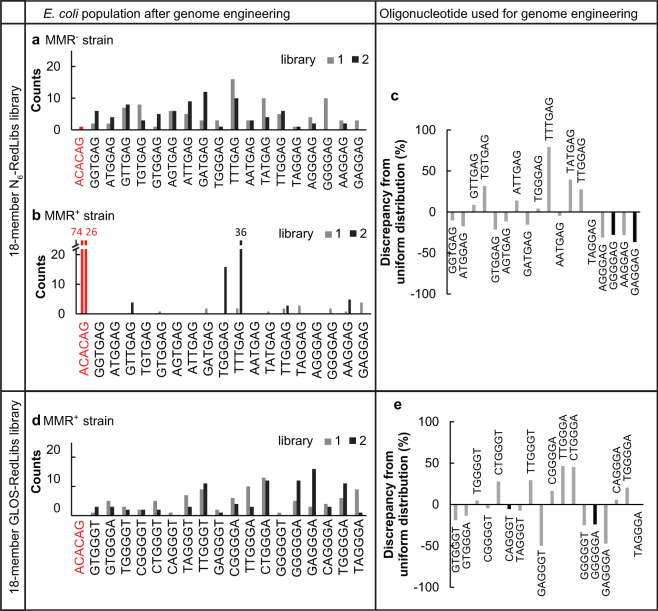
Construction of 18-members N_6_-RedLibs and GLOS-RedLibs genomic *lacZ* RBS libraries. Randomized RBS sequences were created with N_6_-degeneracy or according to the GLOS rules and subsequently reduced to 18-members libraries with RedLibs to uniformly span the TIR range. Frequencies of the 18 members of the N_6_-RedLibs library in (**a**) the MMR^−^ Ec^-^ strain and (**b**) the MMR^+^ EcNR1 strain. N_6_-RedLibs degenerate oligonucleotide sequence: DDKGAG. (**d**) Frequencies of the 18 members of the GLOS-RedLibs RBS library in the MMR^+^ EcNR1 strain. GLOS-RedLibs degenerate oligonucleotide sequence: BDGGGW. Each library was integrated in duplicate and individual clones were analyzed by Sanger sequencing (n = 96 for each library). Red: wild type *lacZ* RBS sequence. RBS sequences showing indels were excluded. (**c** and **e**) Distribution of sequences in the oligonucleotide pool after chemical synthesis for the oligonucleotides used for the N_6_-RedLibs library (**c**) and GLOS-RedLibs library (**e**). Illumina-based oligonucleotide pool sequencing for the different oligonucleotide libraries. Assuming a uniform distribution, each sequence should be represented in the pool with 5.5%, which correspond to 0% discrepancy from uniform distribution. A discrepancy of 50% corresponds to 2.75% abundance in the oligonucleotide pool. Bars in black indicate oligonucleotides for which no corresponding strains could be recovered in the single library preparations.

Next, we analysed the effect of an active MMR system on RBS engineering if GLOS was not applied. We randomized position −10 to −15 relative to the start of *E. coli*’s *lacZ* open reading frame (ORF) with fully degenerate bases (“N_6_-library”) and obtained the predicted TIRs for each RBS sequence from an RBS calculator^5,30^. We used this dataset as input for the RedLibs algorithm to design an oligonucleotide encoding a smart 18-member *lacZ* RBS library with a TIR distribution as uniform as possible (“N_6_-RedLibs”, Fig. 2). This library was introduced into an MMR^+^ and an MMR^−^ strain (EcNR1 and Ec^-^, respectively) and successful mutagenesis was selected for using CRMAGE (Fig. 2). Sanger-sequencing of 96 randomly selected clones obtained for the MMR^−^ strain showed that in two independent experiments, AR efficiencies of at least 98% were achieved and 16 and 18 of the 18 library members, respectively, were recovered. In contrast, in the MMR^+^ strain an average AR efficiency of only 48% was achieved, and only 5 and 9 out of 18 possible sequences could be recovered from the two experiments. Clearly, the MMR system had substantially reduced the diversity of variants that could be sampled with a given number of analyses and substantially increased the number of wild-type clones to further reduce analysis effectiveness.

While an active MMR system clearly decreases library diversity, it might have beneficial effects with respect to other aspects of sequence integrity. Specifically, chemical oligonucleotide synthesis is error-prone and introduces indels on the genome via MAGE close to the target site^23^. We analysed indel frequency within the genomic sequence equivalent to mutagenic oligonucleotides for the 4 times 96 clones obtained before, and found indels in 16.5% of the MMR^−^ clones versus only 7.5% for the MMR^+^ clones (Supplementary Fig. S3a). In other words, MMR^+^ strains have an improved capacity to remove erroneous oligonucleotides, but not sufficient to compensate for the loss in library diversity. In summary, library-based RBS genome editing in regular, MMR^+^ strains is severely hampered.

For comparison we applied the GLOS rule on the N_6_-library (see above), which left us with three nucleotides instead of four at each position (Fig. 1). This results in 3^6^ = 729 oligonucleotides with a 6 bp mismatch at the position of the RBS (“GLOS library”). This GLOS library was then further reduced using RedLibs to generate a single oligonucleotide encoding an 18-member library with TIRs again distributed as uniformly as possible. The resulting smart library was integrated into the genome of MMR^+^ EcNR1 by CRMAGE again in two independent experiments. We observed an improvement of the AR efficiencies to over 98%, with 16 and 18 of the 18 library members incorporated (Fig. 2d). Clearly, the GLOS rule allows maintaining library diversity and analysis effectiveness.

### Remaining deviations from ideal sequence distributions are partially due to biases in folding energy and chemical synthesis

When we inspected the recovered sequences in more detail, we observed that while we found most or even all of sequences that were expected to be present in the library, the abundances were not uniformly distributed. We therefore investigated some potential reasons that might affect the distribution (summarized in Supplementary Table S1), specifically under- or overrepresentation of a sequence within the oligonucleotide pool after chemical synthesis and different folding energies (∆G) of the oligonucleotides. To start with the latter, unstructured oligonucleotides (with higher folding energies) can hybridize to the target more easily and therefore show higher AR efficiencies^20^. Indeed, analysis of the ∆G values of all library members suggested that oligonucleotides with lower folding energy (−7.03 kcal mol^−1^ and −6.05 kcal mol^−1^) were not integrated as efficiently (0 to 5% abundance in the library) as oligonucleotides with higher folding energy (−4.33 kcal mol^−1^; 0 to 40% abundance) (Supplementary Fig. S4a). These results suggest that ∆G has an influence on the final distribution, and that a higher ∆G is indeed beneficial but not a sufficient requirement for a high AR efficiency.

We next examined whether the composition of the initial pool of chemically synthesized DNA oligonucleotides was a source of bias. One potential reason therefore could be again the ∆G of the oligonucleotide since highly structured oligonucleotides are harder to synthesize. We analysed Illumina next-generation sequencing (NGS) data of the synthesized oligonucleotides to detect bias before genome editing. Even though we found up to a 3-fold difference in the oligonucleotide distribution after NGS, there is no correlation with ∆G (Supplementary Fig. S5a). Next we compared the abundances after chemical synthesis with the Sanger-sequencing data of the strains obtained after genome editing to characterize abundances afterwards (Supplementary Fig. S5b). We could not identify a strong correlation between sequence abundances before and after, but did note that the four sequences that we had not found in the four experiments with N_6_-RedLibs-treated MMR^−^ and GLOS-RedLibs-treated MMR^+^ strains were underrepresented by 5% to 37% in the initial oligonucleotide pool (in an ideal uniform distribution pool of 18 oligonucleotides, each sequence would constitute 5.5% of the population; an underrepresentation of 50% of a particular oligonucleotide for example would therefore correspond to an overall abundance of 2.75%). Similarly, the sequences that were recovered most frequently (N_6_-RedLibs library and MMR^−^ strain: TTTGAG; GLOS-RedLibs library and MMR^+^ strain: CTGGGA) were 79% and 45% overrepresented in the pool (Fig. 2c,e). Taken together, the composition of the oligonucleotide library after synthesis as well as oligonucleotide folding energies probably contributed to some extent to the absence or presence of sequences after genome editing.

In conclusion, by including GLOS into the design of a smart RBS library AR efficiency after CRISPR/Cas9-counterselection can be improved from 48% to 98% in an MMR^+^ strain, and the library coverage from 39% to 94%. Looking at a sample of roughly 200 clones (for an 18-membered library), the entire functional space can be covered with reasonable certainty. Careful analysis suggests that deviations from an ideal distribution of sequences after genome editing can be linked to some extent to external factors (such as oligonucleotide selection and/or folding energy and synthesis).

### Application of GLOS to tuning the riboflavin pathway

To test the efficiency of the GLOS-RedLibs strategy on a pathway rather a single gene, we chose riboflavin production in *E. coli* (Supplementary Fig. S2a). All genes are endogenous to *E. coli*, and the product is secreted and easily quantifiable by absorbance or fluorescence measurement in the supernatant^31^, which makes it a suitable model pathway. To identify bottlenecks in the riboflavin production pathway we first analysed the riboflavin production levels when separately overexpressing *ribA, ribB, ribC, ribD*, and *ribE* from low copy plasmids controlled by an inducible promoter (Supplementary Fig. S2b). In case of *ribB*, the genomic negative feedback loop (riboregulator)^32^ is not included on the plasmid. Indeed, overexpression of *ribA*, *ribB*, or *ribC* led to 2-, 3- and 2-fold increased levels of secreted riboflavin, respectively. We chose *ribA* and *ribB* for further experiments.

### Implementation of a GLOS-RedLibs RBS library for ribA

**Figure 3:**
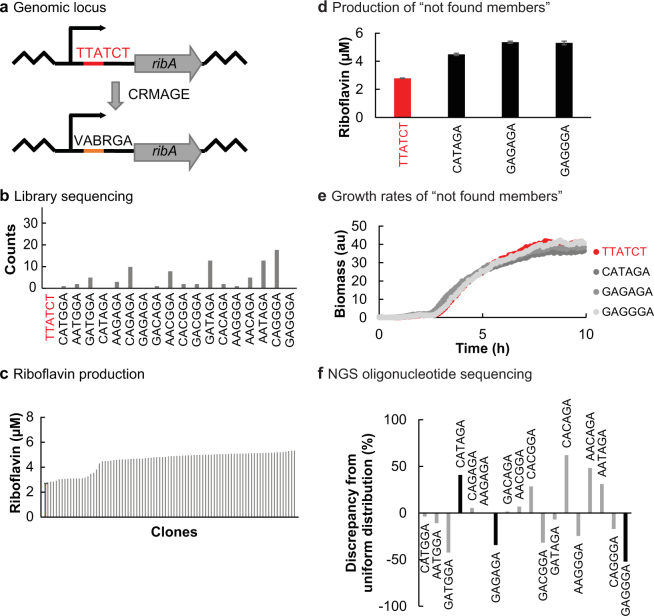
Genomic integration of a chromosomal 18-members GLOS-RedLibs *ribA* RBS library into an MMR^+^ strain. (**a**) Schema of the reduced *ribA* RBS library construction. Upper: wild type *ribA* RBS sequence; lower: GLOS-RedLibs degenerate *ribA* RBS sequence. (**b**) Library clones were analyzed by Sanger sequencing (n = 88). The 18 possible RBS sequence variations (grey) as well as the wild type RBS sequence (red) are listed in ascending order of their predicted TIR. RBS sequences showing indels were excluded from analysis. Therefore, only 86 of 88 sequenced clones are displayed. (**c**) Riboflavin production of all isolated RBS library member strains (n = 88, grey, single measurements) compared to wild type (red, in triplicate, mean ± standard deviation). (**d-e**) Analysis of the separately constructed *ribA* library members CATAGA, GAGAGA, GAGGGA not found in the sequenced library clones in b (**d**) Riboflavin production of the strains compared to wild type (wt, red), values in triplicate are mean ± standard deviation. (**e**) Growth curves of the corresponding strains in LB medium at 30 °C (n = 3). (**f**) Distribution of sequences in the oligonucleotide pool after chemical synthesis. Illumina-based oligonucleotide pool sequencing for the oligonucleotide used to construct this library. Bars in black indicate oligonucleotides for which no corresponding strains could be recovered during library-based genome editing.

We designed a GLOS-RedLibs RBS library for *ribA*, targeting positions −11 to −16 relative to the start of the *ribA* ORF, and integrated it into an MMR^+^ strain (Fig. 3a). We sequenced 88 clones and observed over 98% AR efficiency with 15 of the 18 library members incorporated (Fig. 3b). As previously observed, we found a higher folding energy of the oligonucleotide to be beneficial for the AR efficiency (Supplementary Fig. S4c). In addition, comparison of sequence abundances in the oligonucleotide pool before and in the *E. coli* population after genome editing showed that two of the three missing sequences were already underrepresented before editing (40% and 52%, Fig. 3f). However, in contrast to the earlier experiments with *lacZ*, *ribA* is an essential gene, and therefore clones with a change in TIR might have growth advantages or disadvantages that could have contributed to a non-ideal library sequence distribution in the analysed population. To explore this further, we individually integrated the three missing sequences into the *E. coli* genome in front of *ribA* and determined riboflavin production levels and growth curves. Since all sequences could be incorporated and did not affect growth (Fig. 3e), we exclude differences in growth behaviour as a reason for the fact that they were missing before, and conclude that the imbalance of sequence distribution already before genome editing was the most important source of library bias.

The 88 clones of the library plus the parental clone were examined for riboflavin production (Fig. 3c). The levels of secreted riboflavin varied by a factor of 2.5. When we separately looked at the riboflavin production level of the three strains that were not included in the first library, their levels did not diverge from the overall library range (Fig. 3d). We chose the clone (AACAGA) with the highest riboflavin production (2.5-fold over parent) for further analysis. Notably, although our GLOS-RedLibs library has a reduced potential sequence space compared to an N_6_-RedLibs library (due to the initial reduction in candidate sequences by applying GLOS), we were able to achieve the expression level observed with plasmid-based overexpression (Supplementary Fig. S2).

### Combination of deletion and library insertion

**Figure 4:**
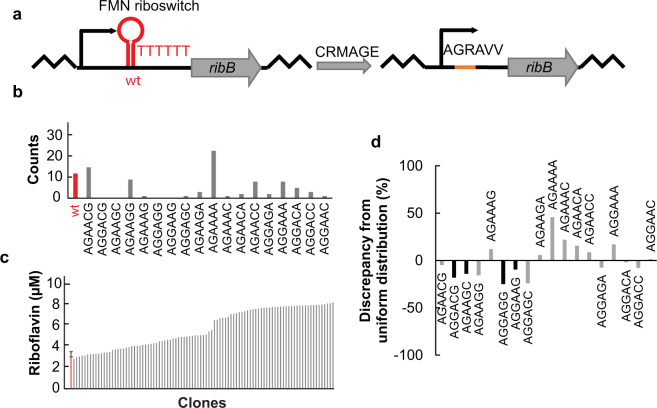
Genomic integration of a chromosomal 18-members GLOS-RedLibs ribB RBS library and FNM riboswitch removal in an MMR^+^ strain. (**a**) Schema of the GLOS-RedLibs ribB RBS library construction. Left: FMN riboswitch and wild type ribB RBS sequence; right: degenerate ribB RBS sequence after GLOS treatment. Note that the riboswitch is supposed to be eliminated concomitantly with the RBS modification step. (**b**) Library clones were analyzed by Sanger sequencing (n = 96). The 18 possible RBS sequences after genome editing (grey) as well as the wild type context (red) are listed in ascending order of their predicted TIR, except wild type since the TIR including the riboswitch cannot easily be estimated. RBS sequences showing mutations were excluded. Therefore, only 94 of 96 sequenced clones are displayed. (**c**) Riboflavin production of all isolated RBS library member strains (n = 96, grey, single measurements) compared to wild type (red, in triplicate, mean ± standard deviation). (**d**) Distribution of sequences in the oligonucleotide pool after chemical synthesis. Illumina-based oligonucleotide pool sequencing for the oligonucleotide used to construct this library. Bars in black indicate oligonucleotides for which no corresponding strains could be recovered during library-based genome editing.

Next, we tested whether the efficient introduction of broad sequence variation by our GLOS method could be implemented in more complex editing processes, specifically if combined with introducing a deletion in the same step. Therefore, we designed an oligonucleotide to delete the FMN riboregulator (220 bp) in front of *ribB* and to concomitantly encode an GLOS-RedLibs RBS library targeting positions −5 to −11 relative to the start of the *ribB* ORF, and integrated this library into MMR^+^ EcNR1 (Fig. 4a). We sequenced 96 of the recovered clones, and observed an AR efficiency of 86% with 14 of 18 possible library members incorporated (Fig. 4b). Similar to *lacZ* and *ribA*, the sequences overrepresented in this set of strains had higher folding energies for the oligonucleotide (Supplementary Fig. S4d) and oligonucleotide sequencing showed that all sequences not found in 96 sequenced clones (AGGAAG, AGGAGG, AGAAGC, AGGACG) had already been underrepresented (10% to 25%) in the oligonucleotide pool (Fig. 4d).

**Figure 5:**
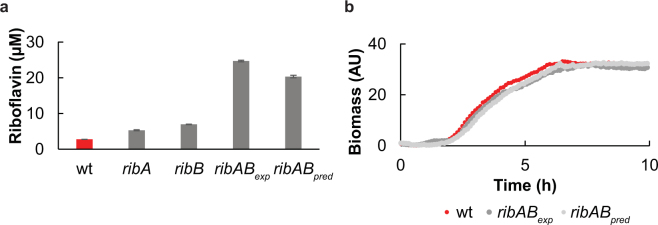
Riboflavin production of *ribAB* double mutants. (**a**) The *ribA* RBS sequence of the highest producer of the *ribA* library (AACAGA) and the *ribB* RBS sequence of the highest producer of the *ribB* library (AGGACA) were combined in one strain by CRMAGE (*ribAB*_*exp*_), and riboflavin production was measured. In comparison a strain with the two strongest predicted RBS in the libraries was created and measured (*ribAB*_*pred*_). Values are in triplicate, mean ± standard deviation. (**b**) Growth curves of all three strains in LB medium at 30 °C (n = 3).

Screening for riboflavin production levels among the recovered strains, we found clones producing up to 2.6-fold more riboflavin than the wild type, indicating that the library nearly covered the riboflavin production range (1- to ~3-fold wild type) expected from the initial plasmid-based overexpression experiments. Production levels of the best producer (AGGACA) were verified in triplicate (Fig. 5a). We conclude that complex modifications like library-based RBS modification with concomitant deletion is possible. However, since the AR efficiency drops in this case, more clones need to be screened to cover the library.

### Combination of experimentally optimized RBSs leads to higher riboflavin production levels than the combination of RBSs that are predicted to show highest TIRs

Since *ribA* and *ribB* are both on the negative strand on replichore 1 and 2, respectively, their RBSs cannot be simultaneously mutated with high efficiency. Therefore, to further improve riboflavin production we combined the RBSs in front of *ribA* and *ribB* found in the respective best producer strains. We also constructed a strain that contained in front of *ribA* and *ribB* the two RBSs that were predicted to show the highest TIRs after GLOS-RedLibs library construction (GAGGGA for *ribA* and AGGAAC for *ribB*). We found that the strain containing the optimized RBSs showed a 9.3-fold increase in riboflavin production over wild type, while the strain containing the RBSs with the highest predicted TIRs showed only a 6.7-fold increase in production (Fig. 5a). Neither mutant exhibited a growth defect when compared to the wild type (Fig. 5b). Even though we realize that this does not constitute a full analysis, we note that combining RBSs that were experimentally optimized results in our case in a better productivity than combining RBSs that were predicted to have the highest TIRs. This confirms the added value of a library-based optimization approach compared to merely combining modifications that produce optimal results when looked at in isolation.

### The engineered MMR^+^ strain has a strongly improved mutational profile

Finally, in order to confirm our original argument that the frequency of undesired mutations is much lower after MAGE-based genome editing in an MMR^+^ than in an MMR^−^ strain, we re-constructed the different RBSs in front of *ribA* and *ribB* and the deletion of the riboregulator in front of *ribB* in an Ec^−^ strain, in which we had previously inactivated the *mutS* gene in two additional MAGE cycles. This way, both strains had undergone the same number of CRMAGE cycles (amounting to eight transformations per strain). The MMR^−^ strain had undergone in addition two transformations to inactivate *mutS*, which is a necessary prerequisite to inactivate the MMR system. Therefore, analysing the genomic sequence of the two strains for off-target mutations should accurately reflect the impact of engineering in an MMR^−^ background. We performed Illumina-based genome sequencing on both strains and compared the sequences to the parental EcNR1 strain. Indeed, the MMR^+^ strain exhibited only 4 off-target mutations, while the MMR^−^ strain exhibited 57 off-target mutations (Supplementary Fig. S1 and Supplementary Table S5).

## Conclusions

The GLOS rule presented here enables fast and efficient construction of smart RBS libraries in MMR^+^ strains while reducing the amount of off-target effects and retaining a higher tendency to repair oligonucleotide-induced indels. Moreover it was previously shown that an active MMR system leads to higher killing efficiency for the CRISPR/Cas9 counter selection^33^. We show that complex modifications such as RBS library construction with simultaneous sequence deletion can be performed in a single step with high AR efficiencies, demonstrating the potential of this method.

In terms of drawbacks, it should be noted that due to the requirement of a 6 bp mismatch it might be that the covered TIR range is not maximal, especially if the targeted Shine-Dalgarno sequence is already similar to the optimal consensus sequence and therefore predicted to be very efficient. In such a case, the members of a GLOS-RBS library might suffer disproportionally much from a 6 bp mismatch. However this can be counteracted by slightly shifting the target region for randomization towards the start codon. Moreover, we believe that the maximal TIRs are unlikely to be required for most applications. Very high TIR values often do not lead to higher activities for various reasons, including metabolic burden or translation being no longer rate-limiting^3^. Furthermore, we argue that this limitation is a reasonable trade-off given that our method drastically reduces off-target mutations and thus might become valuable to many industrial production strains.

It should be mentioned that the recent combination of homologous recombination and CRISPR/Cas9 counterselection represents a different approach to apply library strategies to chromosomal targets^34,35^, which circumvents the issue of library bias due to an active MMR system altogether. However, it remains for now unclear which alternative biases are introduced by replacing oligonucleotide-directed mutagenesis with homologous recombination. In the present method, the different influences have been carefully characterized and it is clear that efficient library strategies can be easily implemented.

Finally, we demonstrated the functionality of this method on *E. coli* but it could be applied to any bacteria which have a similar MMR system. We therefore conclude that GLOS is a helpful tool for many kinds of genomic RBS optimization applications.

## Methods

### Chemicals and media

Restriction enzymes, *Taq* polymerase, Q5 high-fidelity DNA polymerase and T4 DNA ligase were obtained from New England Biolabs (BioConcept AG, Allschwil, Switzerland) and used according to the manufacturer’s instructions. Chemicals were purchased in the highest purity available from Sigma-Aldrich (Buchs, Switzerland) or BD Bioscience (Allschwil, Switzerland). Low salt Difco Luria broth base, Miller (LB Miller) was used for all CRMAGE experiments, where required supplemented with chloramphenicol at 20 µg mL^−1^, kanamycin at 50 µg mL^−1^, streptomycin 50 µg mL^−1^, or carbenicillin at 50 µg mL^−1^ for antibiotic selection. Sucrose was added to a final concentration of 5 g L^−1^ for selection of loss of plasmid pSEVA431_SacB. Medium M9 GCA contained 1× M9 salts^36^, 10 mg L^−1^ thiamine, 2 mg L^−1^ biotin, 0.5 g L^−1^ glucose and 1 g L^−1^ casamino acids. Desalted oligonucleotides were purchased from Sigma-Aldrich (Haverhill, UK) or Microsynth (Balgach, Switzerland). MAGE oligonucleotides contained four phosphorothioated bases at the 5′-end.

### Strains and plasmids

Strains and plasmids used in this study are shown in Supplementary Table S2. The gRNAs for the CRISPR Cas9 system were designed and checked for off-target effects with the web tool COD (Cas9 Online Designer, cas9.wicp.net;^37^). They were inserted into the plasmid pCRISPR according to the Addgene protocol^38^. Shortly, pCRISPR_X plasmids were constructed by digestion of pCRISPR with *Bsa*I followed by insertion of a double stranded linker assembled from oligonucleotide pairs recruited from primers 11–14. Plasmid pSEVA431_SacB was constructed by restriction digest-based cloning. The *sacB* gene including a constitutive promoter was amplified from plasmid pKO3 and *Hind*III and *Eco*RI restriction sites were included by PCR (Q5 polymerase, primer 23 + 24, see Supplementary Table S3). The PCR was done as follows: step 1: 30 s at 95 °C; step 2: 10 s at 95 °C; step 3: 30 s at 50 °C; step 4: 90 s at 72 °C; repeat steps 2 to 4 25 times; step 5: 2 min at 72 °C and storage at 8 °C. The PCR fragment was purified and digested with *Eco*RI and *Hin*dIII and ligated into equally digested pSEVA431. For the plasmids containing the riboflavin genes, the genome of *E. coli* MG1655 was used as a template for the genes encoding the biosynthetic pathway enzymes RibA, RibB, RibC, RibD, and RibE. Each of the *rib* genes was cloned into a separate operon as a transcriptional fusion with the gene for the red fluorescent protein mKate2^39^ downstream of the rib gene. The operons had a common structure where the *rib* genes were fused to a 5′ region containing a cloning site (underlined), and the *rib* RBS sequence (bold) (TCTAGAAATAATTTTGTTTAACTTTAA**GAAGGAGA**TATAAGCTT). Between the two genes, an intergenic region (TAATAAGCTAGAAATAATTTTGTTTAACTTTAA**GAAGGAGAT**ATAAGCTT) containing the *mkate2* RBS sequence (bold) was integrated. After the *mkate2* gene, a 3’ region (TAGTAAGCTAGCTCGAATTC) containing a cloning site (underlined) was inserted. To express the operons, we constructed vector pSEVA261_P_tet_, which allows induction of gene expression via the tet-system in response to anhydrotetracycline (aTc). It was constructed by isolation of the P_tet_ promoter together with *tetR* from plasmid pAB92 by restriction digest with *Spe*I and *Eco*RI and ligation into plasmid pSEVA261, linearized with the same enzymes. Integration of the operons as ribX-mKate2 fragments into pSEVA261_P_tet_ produced plasmids pSEVA261_ribX, where X = A, B, C, D, or E.

### *In silico* RBS library preparation

For all involved genes, the nucleotides of the RBS were identified and fully diversified (N_6_) for the N_6_-libraries. For the GLOS-libraries a partially degenerated sequence was identified so that each sequence in the library is a 6-bp mismatch to the wild-type sequence. Effectively, this means at each position we allowed all bases except for the original one – e.g., if the original sequence was an A the randomization would include a T, C and G at this positon. Appling this rule at 6 consecutive positions ensured that a 6 bp mismatch was created for each library member. The fully degenerate sequence, and the partially degenerate sequence that was designed according to GLOS rules (see above), were submitted to the RBS Calculator version 1.0 in the “Predict: RBS Library” mode^5^ (salislab.net/software) in order to calculate TIR values. For each gene, this data set was then used as input for the RedLibs algorithm version 1.1 as described in the documentation provided with the algorithm’s script (Supplementary file S1; github.com/dgerngross/RedLibs). The RedLibs algorithm was set to generate reduced libraries of 18 sequences whose predicted TIRs distribute as uniformly as possible between the minimal and maximal value of the input library (Supplementary Table S4). The resulting reduced degenerate DNA sequences (one for each gene) were used to design MAGE oligonucleotides (oligonucleotide 1–8, Supplementary Table S3).

### Chromosomal integration: CRMAGE

*E. coli* (strains EcNR1 or Ec^−^, each transformed with plasmid pCas9) were grown in 3 mL of LB Miller supplemented with chloramphenicol at 32 °C until an OD_600_ of approximately 0.6 was reached. Cells were heat-shocked for 15 min at 42 °C to induce production of the Red Beta protein. The induced cells were made electrocompetent by washing 3 times with 1 mL of ice cold water. Then, each oligonucleotide was added to the cells to a final concentration in the cuvette of 2 µM and the cells were electroporated (1 mm gap cuvettes; Cell Projects, Harrietsham, United Kingdom; 1.8 kV and 4–6 ms pulse). Cells were recovered by addition of 3 mL of fresh LB Miller medium supplemented with chloramphenicol for one further CRISPR/Cas9 assisted MAGE cycle. For the second cycle cells were electroporated with 2 µM oligonucleotide and 50 to 100 ng of the pCRISPR plasmid. Cells were incubated for recovery at 32 °C, after 1 h kanamycin was added for selection of pCRISPR, and the incubation was continued overnight. On the next day, cells were plated on selective medium plates with kanamycin and chloramphenicol. Clones were analysed by colony PCR (Multiplex PCR kit; Quiagen, Hombrechtikon, Switzerland). AR efficiency is defined by the ratio of number of mutants and number of total clones. PCR primers (15–20) are listed in Supplementary Table S3. The PCR program was as follows: step 1: 15 min at 95 °C; step 2: 30 s at 95 °C; step 3: 30 s at 50 °C; step 4: 60 s at 72 °C; repeat steps 2 to 4 30 times; step 5: 10 min at 72 °C and storage at 8 °C. To perform the next round of CRMAGE, the strain was cured from pCRISPR by transformation with pSEVA431_SacB that can be selected for by streptomycin. Due to incompatibility of the plasmid origins of replication, pCRISPR was lost during streptomycin selection. Afterward, the strain was grown without antibiotic, and cells that had lost pSEVA431_SacB were selected on medium containing sucrose. Loss of pSEVA431_SacB was confirmed by loss of the antibiotic resistance.

### Sanger sequencing

A PCR on the genomic regions of interest was performed with *Taq* DNA polymerase (primer 17–22 see Supplementary Table S3). The PCR program had the following steps: step 1: 5 min at 95 °C; step 2: 30 s at 95 °C; step 3: 30 s at 50 °C; step 4: 30 s at 68 °C; repeat steps 2 to 4 28 times; step 5: 10 min at 68 °C and storage at 8 °C. PCR products were sequenced by GATC (Konstanz, Germany) or Microsynth (Balgach, Switzerland).

### Next generation sequencing (NGS)

To analyse the off-target effects caused by the MMR^−^ strain, we sequenced the genomes of EcNR1, Ec^−^, Ec^+^ribAB and Ec^−^ribAB on an Illumina MiSeq platform (Illumina RTA Version: 1.18.54, Sequencer: GFB MiSeq, Run type: PE-250). Whole genome data were analysed with the software breseq version 0.26.0^40^. In order to investigate the composition of the oligonucleotide libraries, ssDNA was filled up by a primer extension reaction with one primer (primer 25–27 see Supplementary Table S3). Sequencing adapters were PCR free ligated to the dsDNA fragments by the Hyper Prep Kit (KAPA Biosystems, Wilmington, US) and sequenced by Illumina MiSeq as described above. Oligonucleotide sequencing data were analysed by an in-house developed software for NGS data analysis (S. Schmitt, manuscript in preparation).

### Riboflavin quantification

Strains were incubated in 96-deep well plates (System Duetz, EnzyScreen, Haarlem, Netherlands) for 24 h at 30 °C in M9 GCA medium. Riboflavin was quantified in the cell-free supernatant using an Infinite M1000 PRO microplate reader (TECAN, Maennedorf, Switzerland) measuring fluorescence (excitation wavelength 440 nm and emission wavelength 535 nm). Additionally, the OD_600_ of the culture was measured.

### Growth curves

Growth curves were analysed in 1 mL LB medium at 30 °C and shaking at 800 rpm using a BioLector (m2p-labs GmbH, Baesweiler, Germany) device in a 48-well FlowerPlate (m2p-labs GmbH, Baesweiler, Germany).

### Data availability statement

All data generated or analysed during this study are included in this published article (and its Supplementary Information files), the algorithm’s script used in this published article is provided on github.com/dgerngross/RedLibs.

## Electronic supplementary material

Supplementary Figures and Tables

Supplementary file S1
