# Supplementary material for: Efficient engineering of chromosomal ribosome binding site libraries in mismatch repair proficient Escherichia coli
Source: Sci Rep. 2017 Sep 26;7:12327. doi: 10.1038/s41598-017-12395-3 (PMC5615074; doi:10.1038/s41598-017-12395-3)
Supplement: Supplementary file 1 — Supplementary Figures and Tables [file 41598_2017_12395_MOESM1_ESM.pdf]

Efficient engineering of chromosomal ribosome binding site libraries in mismatch repair proficient *Escherichia coli*

Sabine Oesterle[1], Daniel Gerngross[1], Steven Schmitt[1], Tania Michelle Roberts[1], and Sven Panke[1]*

Supplementary material

Supplementary Table 1: **Possible sources of bias leading to non-uniform distribution of library members.** Occurrence of biases depends on genetic configuration of the host strain (MMR^- or MMR^+) and the design strategy (N_6-RedLibs or GLOS-RedLibs) for the supplied oligonucleotide pool during CRMAGE.

	N_6-RedLibs (0-6 bp mismatch)		GLOS-RedLibs (6 bp mismatch)
	MMR^-	MMR^+	MMR^+
DNA synthesis-derived bias in oligonucleotide pool	yes	yes	yes
AR efficiency bias due to			
- different length of the mismatch	yes	yes	no
- folding energy of the oligonucleotide	yes	yes	yes
- generation at which the oligonucleotide is integrated	yes	yes	yes
CRISPR/Cas9 counterselection			
- inactivation due to mutation of the components	yes	no	no
- bias in escape rate due to length and position of the mismatch between gRNA and target	yes	yes	no
MMR bias due to length or type of mismatch	no	yes	no
Survival rate bias due to essentiality or metabolic burden	yes	yes	yes

Supplementary Table 2: Strains and Plasmids used in this study.

**Strains**	**Relevant genotype**	**Source/reference**
BW23474	*E. coli* Δ(*argF-lac*)*169*, Δ*uidA4*::*pir-116*, *recA1*, *rpoS396*(Am), *endA9*(del-ins)::*FRT*, *rph-1*, *hsdR514*, *rob-1*, *creC510*	[1]
Top10	*E. coli F*- mcrA Δ(mrr-hsdRMS-mcrBC) φ80lacZΔM15 ΔlacX74 nupG recA1 araD139 Δ(ara-leu)7697 galE15 galK16 rpsL(Str^R) endA1 λ^-	Invitrogen
MG1655	*E. coli F-, lambda-, rph-1*	Coli Genetic Stock Center (CGSC)
EcNR1	*E. coli* MG1655 Δ(*ybhB-bioAB*)::[λcI857 N(*cro-ea59*)::*tetR-bla*]	Addgene #26930[2]
Ec^-	EcNR1 translational knockout of *mutS*	this study
Ec^+LacZ_Red	EcNR1 with a RBS variant (DDKGAG) for *lacZ*	this study
Ec^-LacZ_Red	Ec- with a RBS variant (DDKGAG) for *lacZ*	this study
Ec^+LacZ_GLOS	EcNR1 with a RBS variant (BDGGGW) for *lacZ*	this study
Ec^+ribA	EcNR1 with a RBS variant (VABRGA) for *ribA*	this study
Ec^+ribB	EcNR1 with a RBS variant (AGRAVV) for *ribB* and deletion of the riboregulator of *ribB*	this study
Ec^+ribAB_{exp}	EcNR1 with RBS variant (AACAGA) for *ribA* and (AGGACA) for *ribB* and deletion of the riboregulator of *ribB*	this study
Ec^+ribAB_{pred}	EcNR1 with RBS variant (GAGGGA) for *ribA* and (AGGAAC) for *ribB* and deletion of the riboregulator of *ribB*	this study
Ec^-ribAB	Ec^- with a RBS variant (AAGAGA) for *ribA* and (AGGACA) for *ribB* and deletion of the riboregulator of *ribB*	this study
**Plasmids**		
pCas9	Bacterial expression of Cas9 nuclease, chloramphenicol resistance, p15A ori	Addgene #42876[3]
pCRISPR	A crRNA expression plasmid for targeting a specific sequence of choice, kanamycin resistance, pBR322 ori	Addgene #42875[3]
pCRISPR_lacZ	pCRISPR with gRNA targeting the RBS of *lacZ*	this study
pCRISPR_ribA	pCRISPR with gRNA targeting the RBS of *ribA*	this study
pCRISPR_ribB	pCRISPR with gRNA targeting the RBS of *ribB*	this study
pSEVA261	p15A ori and kanamycin resistance	pSEVA collection[4]
pAB92	pBR322 ori, ampicillin resistance gene, P_{tet}, TetR	[5]
pSEVA261_P_{tet}	pSEVA261 with P_{tet} and TetR	this study
pSEVA261_mKate	pSEVA261_P_{tet} plasmid with *mKate2* under control of a P_{tet}	this study
pSEVA261_ribA	pSEVA261_P_{tet} plasmid with *ribA* (EcoGene accession number: EG11331) and *mKate2* under control of a P_{tet}	this study
pSEVA261_ribB	pSEVA261_P_{tet} plasmid with *ribB* (EcoGene accession number: EG10465) and *mKate2* under control of a P_{tet}	this study
pSEVA261_ribC	pSEVA261_P_{tet} plasmid with *ribC* (EcoGene accession number: EG11406) and *mKate2* under control of a P_{tet}	this study
pSEVA261_ribD	pSEVA261_P_{tet} plasmid with *ribD* (EcoGene accession number: EG11321) and *mKate2* under control of a P_{tet}	this study
pSEVA261_ribE	pSEVA261_P_{tet} plasmid with *ribE* (EcoGene accession number: EG11322) and *mKate2* under control of a P_{tet}	this study
pKO3	Plasmid with *B. subtilis* gene *sacB* under control of a P_{const}	[6]
pSEVA431	pBR322 ori, streptomycin resistance	pSEVA collection[4]
pSEVA431_SacB	pSEVA431 plasmid with *sacB* under control of a P_{const}	this study

Supplementary Table 3: Oligonucleotides used in this study.

ID	Name	Sequence (5' to 3')[a]
1	lacZ_RBS	T*G*C*T*TCCGGCTCGTATGTTGTGTGGAATTGTGAGCGGATAACAATTT CDDKGAGGAAACAGCTATGACCATGATTACGGATTCACTGGCC
2	lacZ_RBS_GLOS	T*G*C*T*TCCGGCTCGTATGTTGTGTGGAATTGTGAGCGGATAACAATTT CBDGGGWGAAACAGCTATGACCATGATTACGGATTCACTGGCC
3	ribA_RBS_GLOS	C*C*C*T*TCTCGTTATGGCAAAATAAGCCAATACAGAACCAGCAVABRGA GGAGAATTTCATGCAGCTTAAACGTGTGGCAGAAGCCAAACTG
4	ribB_RBS_GLOS	A*A*A*A*GAGGAAAGTAGCGTCTGATTCATGGTABBTYCTCCTCACTAACT GAGAATAAGCGGATTCACTATAACGCTAATGATTAGCGGCAG
5	ribA_GAGGGA	C*C*C*T*TCTCGTTATGGCAAAATAAGCCAATACAGAACCAGCAGAGAGA GGAGAATTTCATGCAGCTTAAACGTGTGGCAGAAGCCAAACTG
6	ribA_GAGAGA	C*C*C*T*TCTCGTTATGGCAAAATAAGCCAATACAGAACCAGCAGAGGGA GGAGAATTTCATGCAGCTTAAACGTGTGGCAGAAGCCAAACTG
7	ribA_CATAGA	C*C*C*T*TCTCGTTATGGCAAAATAAGCCAATACAGAACCAGCACATGGA GGAGAATTTCATGCAGCTTAAACGTGTGGCAGAAGCCAAACTG
8	ribA_ AACAGA	C*C*C*T*TCTCGTTATGGCAAAATAAGCCAATACAGAACCAGCAAACAGA GGAGAATTTCATGCAGCTTAAACGTGTGGCAGAAGCCAAACTG
9	pCRISPR_lacZ _fw	[PHOS]AAACTGGAATTGTGAGCGGATAACAATTTCACACG
10	pCRISPR_lacZ _rv	[PHOS]AAAAC GTGTGAAATTGTTATCCGCTCACAATTCCA
11	pCRISPR_ribA_fw	[PHOS]AAACAAAATAAGCCAATACAGAACCAGCATTATCG
12	pCRISPR_ribA_rv	[PHOS]AAAAC GATAATGCTGGTTCTGTATTGGCTTATTTT
13	pCRISPR_ribB_fw	[PHOS]AAACATCAACTCAGTTGAAAGCCCGCGAGCGCTTG
14	pCRISPR_ribB_rv	[PHOS]AAAACAAGCGCTCGCGGGCTTTCAACTGAGTTGAT
15	lacZ_RBS_rv	TCATAGCTGTTTCCTGTGT
16	ribA_RBS_rv	GCTGCATGAAATTCTCCAGATAA
17	ribB_rv	ACACGTTCGAAAGGCGTAC
18	lacZ_RBS_fw	GTTGGCCGATTCATTAATG
19	ribA_RBS_fw	ACTGCGGTACGTCTGG
20	ribB_fw	TTGCTCAATTTTTTTCGGG
21	lacZ_RBS_seq_rv	GGACGACGACAGTATC
22	ribA_RBS_seq_rv	GCTTCGAGCTGGAAGC
23	SacB_fw	ATATATAAGCTTCACATATACCTGCCGTTCAC
24	SacB_rv	ATATATGAATTCTTATTTGTTAACTGTTAATT
25	Fill_up_lacZ	GGCCAGTGAATCC
26	Fill_up_RibA	CAGTTTGGCTTCTGC
27	Fill_up_RibB	CTGCCGCTAATCAT

a Phosphorothioated bases are indicated with an asterisk after the base.

Supplementary Table 4: **Different sequences of each RBS library and their predicted TIR and ΔG of the secondary structure formed by the chemically synthesized oligonucleotide.** TIRs are predicted by the Salis RBS Calculator version 1.0 in the “Predict: RBS Library” mode [7]. No value for the TIR of RibB wt is given since the riboregulator affects the TIR. Folding energies (ΔG) of all oligonucleotides were calculated on the mFold web server [8].

**LacZ N_6-RedLibs**			**LacZ GLOS-RedLibs**			**RibA GLOS-RedLibs**			**RibB GLOS-RedLibs**		
**Sequence**	**TIR (AU)**	**ΔG (kcal mol^{-1})**	**Sequence**	**TIR (AU)**	**ΔG (kcal mol^{-1})**	**Sequence**	**TIR (AU)**	**ΔG (kcal mol^{-1})**	**Sequence**	**TIR (AU)**	**ΔG (kcal mol^{-1})**
wt	731		wt	732		wt	3		wt	-	
GGTGAG	916	-4.33	GTGGGT	69	-5.47	CATGGA	2358	-10.54	AGAACG	2376	-8.27
ATGGAG	1104	-7.03	GTGGGA	325	-5.1	AATGGA	5544	-9.95	AGGACG	2845	-9.18
GTTGAG	1147	-5.02	TGGGGT	340	-5.47	GATGGA	7263	-9.95	AGAAGC	4265	-9.79
TGTGAG	1256	-5.41	CGGGGT	348	-5.47	CATAGA	10743	-8.49	AGAAGG	5845	-8.20
GTGGAG	1321	-5.1	CTGGGT	359	-5.47	AAGAGA	11915	-8.47	AGAAAG	6997	-8.30
AGTGAG	1510	-6.05	CAGGGT	470	-5.92	CAGAGA	11915	-8.67	AGGAGG	10030	-8.81
ATTGAG	3379	-4.68	TAGGGT	800	-5.47	GAGAGA	13037	-8.57	AGGAAG	13139	-9.53
GATGAG	3379	-4.33	TTGGGT	821	-5.47	GACAGA	13454	-8.27	AGGAGC	15731	-10.50
TGGGAG	3868	-4.69	GAGGGT	881	-5.47	AACGGA	14265	-8.37	AGAAGA	19701	-8.20
TTTGAG	4046	-4.33	CGGGGA	1448	-4.68	CACGGA	16327	-8.27	AGAAAA	23586	-8.20
AATGAG	4046	-4.57	TTGGGA	1880	-4.75	GACGGA	18437	-8.27	AGAAAC	23586	-8.20
TATGAG	7048	-4.33	CTGGGA	1880	-4.33	GATAGA	19285	-8.27	AGAACA	23586	-8.20
TTGGAG	10955	-4.33	GGGGGT	2441	-5.47	CACAGA	22073	-8.27	AGAACC	23586	-8.20
TAGGAG	11563	-4.33	GGGGGA	3227	-4.33	AAGGGA	22407	-8.46	AGGAGA	25808	-8.81
AGGGAG	14351	-4.65	GAGGGA	3375	-4.33	AACAGA	25264	-9.67	AGGAAA	27610	-9.75
GGGGAG	18800	-4.33	CAGGGA	3898	-5.38	AATAGA	31639	-8.37	AGGACA	36992	-8.81
AAGGAG	19843	-4.57	TGGGGA	4227	-4.33	CAGGGA	38975	-8.33	AGGACC	36992	-8.81
GAGGAG	28442	-5.94	TAGGGA	9167	-4.65	GAGGGA	40769	-8.33	AGGAAC	42340	-8.81

Supplementary Table 5: **List of all off-target mutations in Ec[+]ribAB and Ec[-]ribAB** after four CRMAGE cycles and four CRMAGE and two MAGE cycles for deactivation of *mutS*, respectively. Three mutations (*) occurred in the *mutS* deactivation step. Genome sequencing data were analyzed by breseq [9], arrows indicate the orientation of the gene on the genome (→ plus strand and ← minus strand). If the mutation is between two genes (intergenic), both flanking genes are reported. If the gene does not code for a protein the entry in "aa change" is "noncoding". Gene descriptions are taken from input GenBank files by breseq (Deatherage and Barrick).

Ec[+]ribAB

Position	Mutation	aa change	Gene	Description
158,933	A→G	V54A	*pcnB* ←	poly(A) polymerase I
1,588,170	C→T	intergenic	*b1505* ← / ← *b15 06*	putative outer membrane protein, hypothetical protein
3,636,556	G→A	A298T	*pitA* →	low-affinity phosphate transport
4,164,808	G→T	noncoding	*rrsB* →	16S ribosomal RNA

Ec[-]ribAB

Position	Mutation	aa change	Gene	Description
53,484	A→G	S407P	*surA* ←	survival protein
74,110	C→T	G138S	*yabK* ←	putative transport system permease protein
119,651	A→C	D124A	*ampE* →	regulates *ampC*
123,331	C→T	G105G	*aceE* →	pyruvate dehydrogenase (decarboxylase component)
157,118	T→C	intergenic	*yadN*←/←*folK*	putative fimbrial-like protein/ 7,8-dihydro-6-hydroxymethylpterin-pyroph osphokinase
158,672	C→T	G141D	*pcnB* ←	poly(A) polymerase I
166,050	A→G	T441A	*mrcB* →	peptidoglycan synthetase; penicillin-binding protein 1B
182,555	C→T	A37A	*cdaR* →	regulator of D-galactarate, D-glucarate and D-glycerate metabolism
204,986	A→G	Y165C	*rnhB* →	RNAse HII, degrades RNA of DNA-RNA hybrids
273,119	T→C	V345A	*ykfC* →	hypothetical protein
280,733	G→A	R159C	*yagA* ←	hypothetical protein
320,783	G→A	intergenic	*ykgD*→/→*ykgE*	putative ARAC-type regulatory protein/putative dehydrogenase subunit
347,141	A→G	I176T	*prpR* ←	regulator for *prp* operon
512,101	C→T	T101M	*ybaT* →	putative amino acid/amine transport protein
543,006	A→G	S89P	*ylbA* ←	hypothetical protein
664,168	C→T	L82L	*rlpA* ←	a minor lipoprotein
703,366	A→G	D67G	*nagE* →	PTS system, N-acetylglucosamine-specific enzyme IIABC
834,779	T→C	I103I	*ybiB* →	putative enzyme
900,961	C→T	V171V	*artQ* ←	arginine 3rd transport system permease protein
1,113,039*	A→G	D123D	*msyB* ←	acidic protein suppresses mutants lacking function of protein export
1,136,623	T→C	S10S	*flgJ* →	flagellar biosynthesis
1,191,766	T→C	H30R	*ycfC* ←	hypothetical protein
1,275,414	A→G	F476F	*narX* ←	nitrate/nitrate sensor, histidine protein kinase acts on NarL regulator

1,333,276	G→A	intergenic	*cysB→/→acnA*	positive transcriptional regulator for cysteine regulon/aconitate hydrase 1
1,346,830	A→G	L36P	*rnb* ←	RNase II, mRNA degradation
1,374,703	A→G	S219G	*ycjR* →	hypothetical protein
1,416,937*	A→G	L122L	*sieB* →	phage superinfection exclusion protein
1,536,890	T→C	Q1242R	*narZ* ←	cryptic nitrate reductase 2 alpha subunit
1,554,862	G→A	G72R	*osmC* →	osmotically inducible protein
1,620,820	G→A	intergenic	*ydeE→/←ydeH*	putative transport protein/orf, hypothetical protein
1,894,197	G→A	E2K	*yeaB* →	hypothetical protein
1,898,742	T→C	N290D	*yoaE* ←	putative transport protein
1,906,040	$(A)_{7\rightarrow8}$	intergenic	*yobF←/←yebO*	hypothetical protein/hypothetical protein
2,086,286	C→T	A6A	*yeeY* ←	putative transcriptional regulator LYSR-type
2,102,024	repeat region +9 bp	coding	*wbbK* ←	putative glucose transferase
2,503,109	A→G	V154A	*ypdE* ←	hypothetical protein
2,602,988	C→T	A52A	*hyfD* →	hydrogenase 4 membrane subunit
2,757,357	$(A)_{8\rightarrow9}$	coding	*yfjI* →	hypothetical protein
2,768,789	A→G	K112K	*yfjS* →	hypothetical protein
2,770,160	A→G	I6T	*yfjU* ←	hypothetical protein
2,816,972	A→G	intergenic	*serV←/←csrA*	tRNA-Ser/carbon storage regulator
3,076,675	A→G	G69G	*cmtA* ←	PTS system, mannitol-specific enzyme II component, cryptic
3,081,206	T→C	N205S	*speB* ←	agmatinase
3,165,071	T→C	T224A	*ygiS* ←	putative transport periplasmic protein
3,204,250	T→C	D10G	*ygiP* ←	putative transcriptional regulator LYSR-type
3,303,854	A→G	intergenic	*mtr←/ ←deaD*	tryptophan-specific transport protein/inducible ATP-independent RNA helicase
3,359,337	T→C	V47A	*gltF* →	regulator of *gltBDF* operon, induction of Ntr enzymes
3,441,036	T→C	T362A	*prlA* ←	putative ATPase subunit of translocase
3,823,370	T→A	D46E	*recG* →	DNA helicase, resolution of Holliday junctions, branch migration
4,047,248	C→T	R754C	*polA* →	DNA polymerase I
4,047,374	C→T	P796S	*polA* →	DNA polymerase I
4,317,108	C→T	P174P	*phnJ* ←	phosphonate metabolism
4,380,567	$(T)_{7\rightarrow6}$	intergenic	*frdA←/→yjeA*	fumarate reductase, anaerobic, flavoprotein subunit/putative lysyl-tRNA synthetase
4,455,398	G→A	P164L	*yjgA* ←	putative alpha helix protein
4,548,135	A→G	G395G	*gntP* ←	gluconate transport system permease 3
4,581,282*	T→C	E1186G	*hsdR* ←	host restriction; endonuclease R
4,637,628	A→G	G234G	*arcA* ←	negative response regulator of genes in aerobic pathways, (sensors, ArcB and CpxA)

Supplementary Figures:

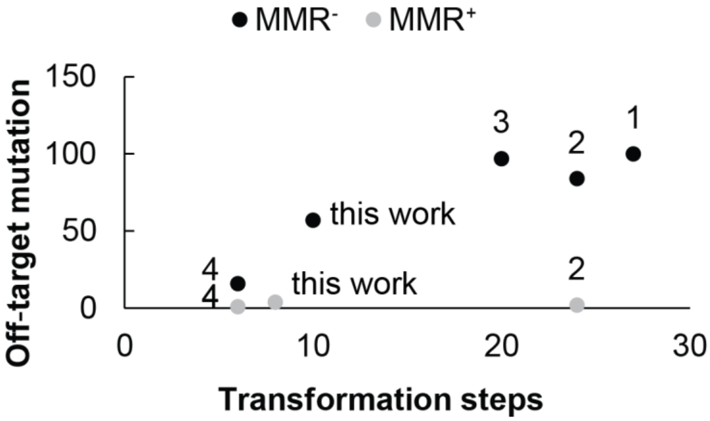

Supplementary Figure 1: **Genomic off-target mutations introduced by MAGE** observed in different MAGE projects taken from literature: 1: [10]; 2: [11]; 3: [12] and 4: [13] in MMR$^-$ (black) and MMR$^+$ (grey), each MAGE cycle is counted as one transformation step. For our work transformation steps are either plasmid or oligonucleotide transformation for Ec$^-$ribAB or Ec$^+$ribAB (four oligonucleotide and four plasmid transformation or six and four, respectively. For Ec$^-$ribAB two additional step were required to deactivate *mutS* by introduction of an early STOP codon).

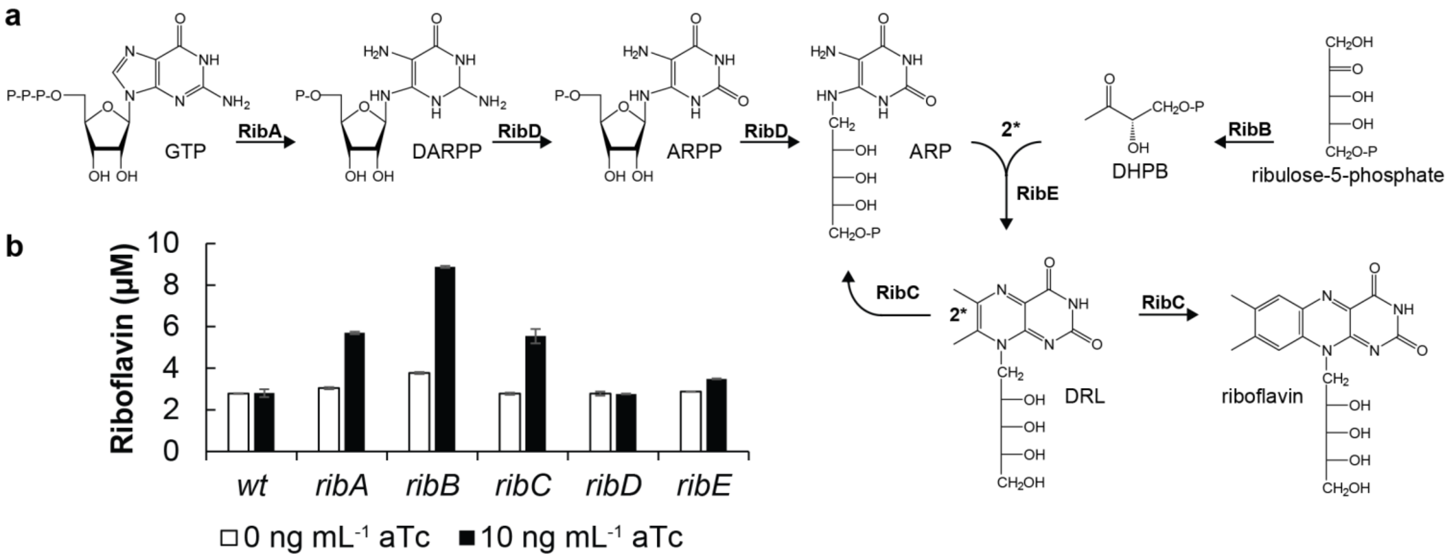

Supplementary Figure 2. **Riboflavin production** (**a**) Pathway of riboflavin synthesis in *E. coli* and involved enzymes. GTP: Guanosine 5'-triphosphate; DARPP: 2,5-Diamino-6-(5-phospho-D-ribosylamino)pyrimidin-4(3H)-one; ARPP: 5-Amino-6-(5'-phosphoribosylamino)uracil; ARP: 5-amino-6-(5'-phospho-D-ribitylamino)uracil; DHPB: L-3,4-dihydroxybutan-2-one 4-phosphate DRL: 6,7-dimethyl-8-(D-ribityl)lumazine. (**b**) Riboflavin production after 24 h at 30°C in the wildtype strain *E. coli* BW23474 carrying an empty expression plasmid (wt) and after plasmid-based overexpression of single riboflavin genes after induction with 0 (white) or 10 ng mL^{-1} (black) of anhydrotetracyclin (aTc). Note that in this setup all *rib* genes are also present and functional at their endogenous chromosomal locus. Values are mean (n = 3) ± standard deviation.

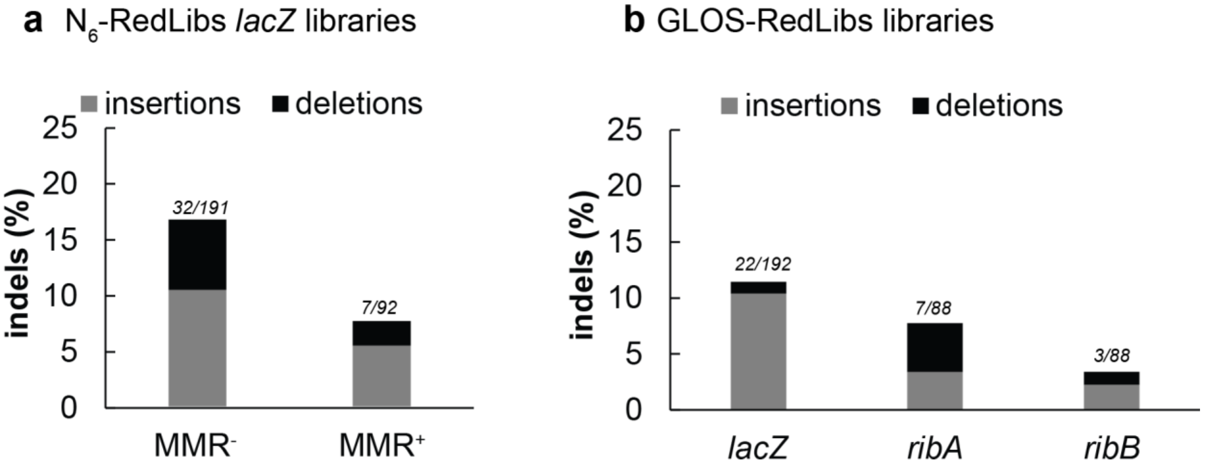

Supplementary Figure 3. **Indels found within the oligonucleotide-covered sequences after genome editing.** Fraction of all library members which have an insertion (grey) or deletion (black) on the genome within the 90 bp oligonucleotide sequence. (**a**) Comparison of the N_6-RedLiba *lacZ* library in an MMR^- and MMR^+ strain. (**b**) Analysis of the GLOS-RedLibs libraries. Frequencies were determined by Sanger sequencing. Numbers indicate absolute numbers, mutants with indels/total. Wild type sequences were excluded from the analysis.

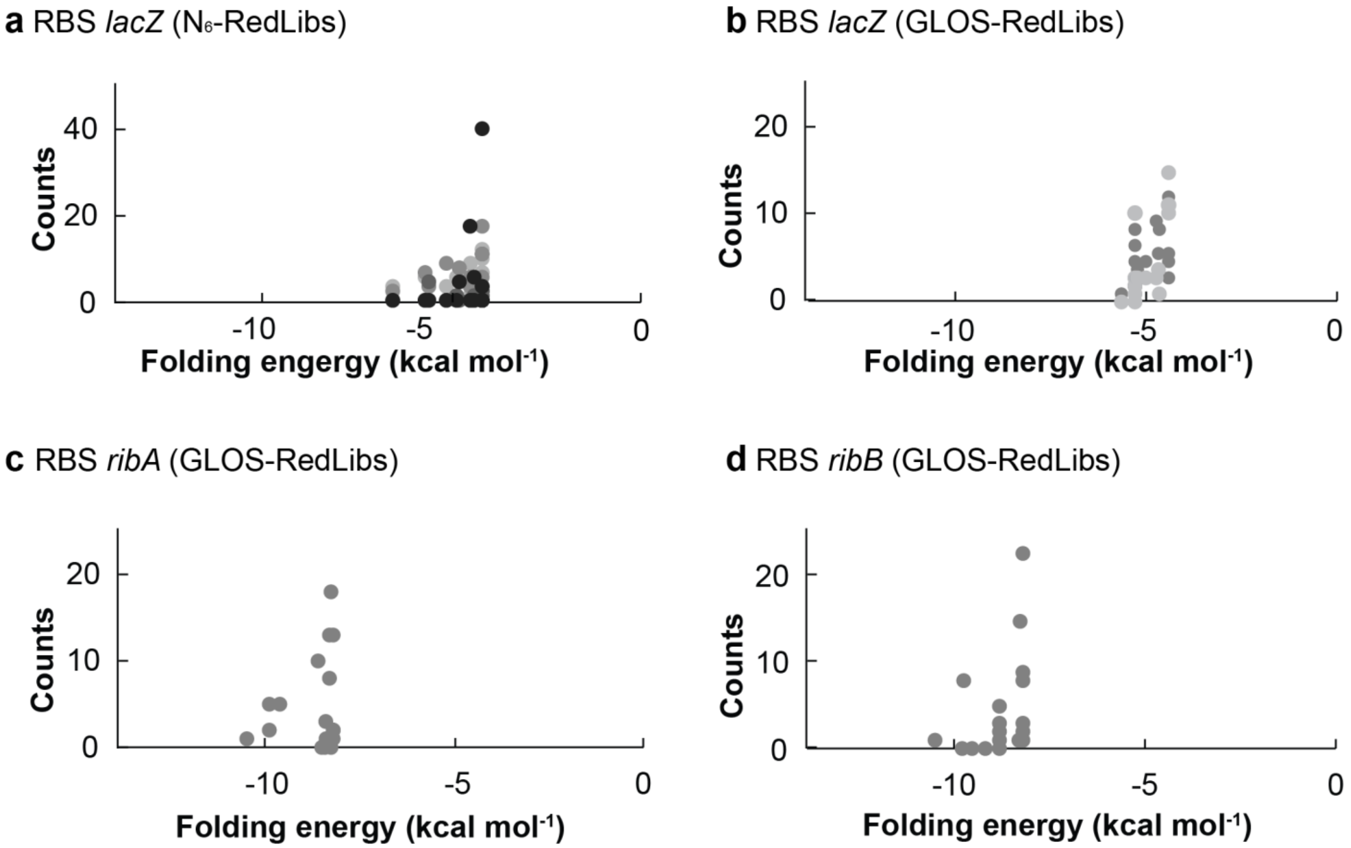

Supplementary Figure 4. **Folding energies (∆G) of all chemically synthesized oligonucleotides present in RBS libraries and their occurrence after genome editing**. Folding energies were calculated (mFold web server[8]) for each of the 18 members of each library and are plotted against the frequency with which a strain was found that had acquired the corresponding change in RBS. Note that those library members for which no corresponding strain could be identified have the count "0". (**a**) Data for the *lacZ* (N_6-RedLibs) library, two experiments in MMR^+ and two experiments in MMR^- strains. Different shades of grey refer to the four different experiments. Compare also Fig. 2a (**b**) Data for the *lacZ* (GLOS-RedLibs) library, two experiments in MMR^+ strain. Different shades indicate the different libraries displayed in Fig. 2abd. (**c**) Data for the *ribA* (GLOS-RedLibs) library, see Fig. 3b. (**d**) Data for *ribB* (GLOS-RedLibs) library see Fig. 4b.

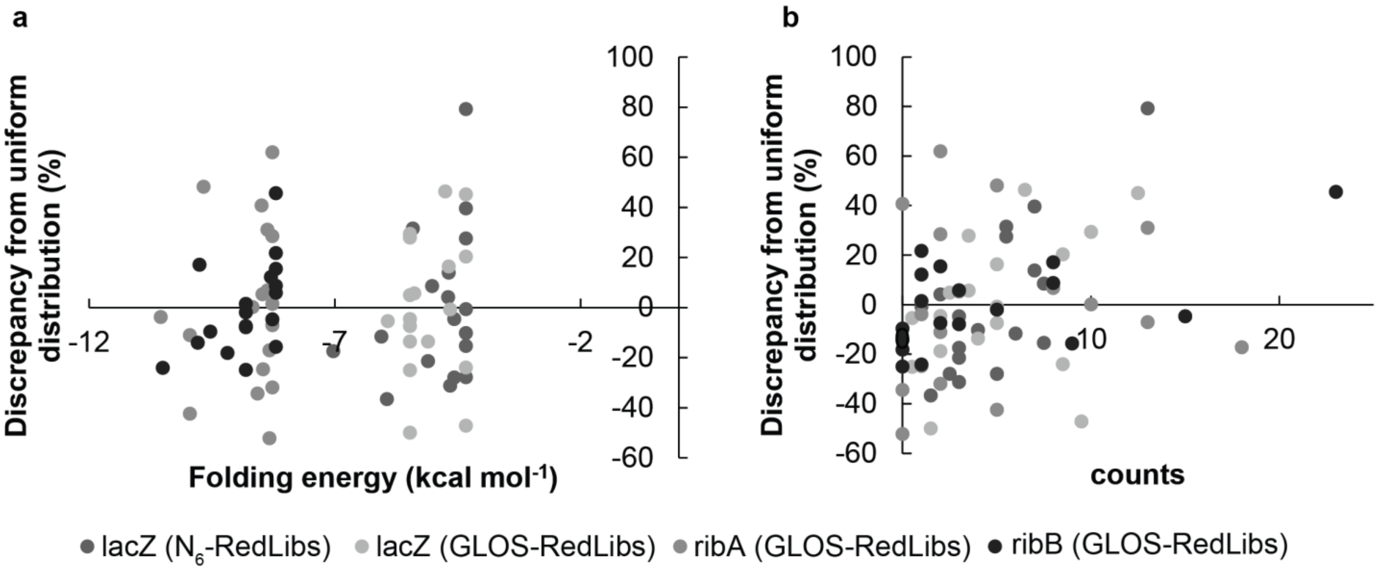

Supplementary Figure 5. **Aspects of sequence distribution.** Relation between the deviation of the abundance a specific oligonucleotide after chemical synthesis from uniform distribution and a) the folding energy (ΔG) of the corresponding chemically synthesized oligonucleotide and (b) the occurrence of the corresponding sequence after genome editing.

Additional references

1. Haldimann, A. & Wanner, B. L. Conditional-replication, integration, excision, and retrieval plasmid-host systems for gene structure-function studies of bacteria. *J. Bacteriol.* **183,** 6384–6393 (2001).

2. Wang, H. H. *et al.* Programming cells by multiplex genome engineering and accelerated evolution. *Nature* **460,** 894–898 (2009).

3. Jiang, W., Bikard, D., Cox, D., Zhang, F. & Marraffini, L. A. RNA-guided editing of bacterial genomes using CRISPR-Cas systems. *Nat. Biotechnol.* **31,** 233–239 (2013).

4. Silva-Rocha, R. *et al.* The Standard European Vector Architecture (SEVA): A coherent platform for the analysis and deployment of complex prokaryotic phenotypes. *Nucleic Acids Res.* **41,** 666–675 (2013).

5. Bosshart, A., Hee, C. S., Bechtold, M., Schirmer, T. & Panke, S. Directed divergent evolution of a thermostable D -tagatose epimerase towards improved activity for two hexose substrates. *ChemBioChem* **16,** 592–601 (2015).

6. Link, A. J. & Phillips, D. Methods for generating precise deletions and insertions in the genome of wild-type *Escherichia coli*: application to open reading frame characterization. **179,** 6228–6237 (1997).

7. Salis, H. M. The ribosome binding site calculator. *Methods Enzymol.* **498,** 19–42 (2011).

8. Zuker, M. Mfold web server for nucleic acid folding and hybridization prediction. *Nucleic Acids Res.* **31,** 3406–3415 (2003).

9. Deatherage, D. E. & Barrick, J. E. Identification of mutations in laboratory-evolved microbes from next-generation sequencing data using breseq. *Methods Protoc. Methods Mol. Biol.* **1151,** 165–188 (2014).

10. Isaacs, F. J. *et al.* Precise manipulation of chromosomes *in vivo* enables genome-wide codon replacement. *Science* **333,** 348–353 (2011).

11. Nyerges, Á. *et al.* A highly precise and portable genome engineering method allows comparison of mutational effects across bacterial species. *Proc. Natl. Acad. Sci.* **113,** 2502–2507 (2016).

12. Nyerges, Á. *et al.* Conditional DNA repair mutants enable highly precise genome engineering. *Nucleic Acids Res.* **42,** e62 (2014).

13. Lennen, R. M. *et al.* Transient overexpression of DNA adenine methylase enables efficient and mobile genome engineering with reduced off-target effects. *Nucleic Acids Res.* **44,** e36–e36 (2016).
